# IQCA-TAVV: To explore the effect of P-selectin, GPIIb/IIIa, IL-2, IL-6 and IL-8 on deep venous thrombosis

**DOI:** 10.18632/oncotarget.20588

**Published:** 2017-08-24

**Authors:** Jianhui Wu, Haimei Zhu, Guodong Yang, Yuji Wang, Yaonan Wang, Shurui Zhao, Ming Zhao, Shiqi Peng

**Affiliations:** ^1^ Beijing Area Major Laboratory of Peptide and Small Molecular Drugs, Engineering Research Center of Endogenous Prophylactic of Ministry of Education of China, Beijing Laboratory of Biomedical Materials, College of Pharmaceutical Sciences of Capital Medical University, Beijing, PR China; ^2^ Department of Biomedical Science and Environmental Biology, Kaohsiung Medical University, Kaohsiung, Taiwan

**Keywords:** deep vein thrombosis, P-selectin, GPIIb/IIIa, cytokines, inflammation

## Abstract

Deep vein thrombosis (DVT) associates with considerable morbidity, functional disability and mortality. Due to the lack of suitable inhibitor the correlation of various factors in DVT onset remains unknown. In this context we analyzed the structure of anti-platelet aggregation agent, P-selectin down-regulator, GPIIb/IIIa down-regulator and anti-inflammatory agent, thereby designed N-(3S-1,2,3,4-tetrahydroisoquinoline-3-carbonyl)- Thr-Ala-Arg-Gly-Asp(Val)-Val (IQCA-TAVV) as an inhibitor of DVT to receive evaluations. The docking predicted that IQCA-TAVV can target P-selectin and GPIIb/IIIa. The UV showed that IQCA-TAVV can act on P-selectin and GPIIb/IIIa. ELISA indicated that IQCA-TAVV concentration dependently inhibited activated platelets to express P-selectin and GPIIb/IIIa, and the minimal effective concentration was 1 nM. IC_50_ of IQCA-TAVV against platelet aggregation induced by arachidonic acid, adenosine diphosphate and platelet activating factor fell within a range of 0.13 nM to 0.30 nM. *In vivo* IQCA-TAVV dose-dependently inhibited venous thrombosis and the minimal effective dose was 1 nmol/kg. On ear edema model the anti-inflammation activity of 10 nmol/kg IQCA-TAVV equaled that of 1.1mmol/kg aspirin. The concentration of IL-2, IL-6 and IL-8 in the serum of the ear edema mice were also significantly decreased by 10 nmol/kg IQCA-TAVV. Even at 1 μmol/kg of dose IQCA-TAVV still did not injure the kidney, the liver, and the nerves of healthy mice. Thereby IQCA-TAVV depicts a relationship of three levels (inhibiting platelet activation, targeting externalized membrane receptor, decreasing serum inflammatory factor) for the down-regulation of P-selectin, GPIIb/IIIa, IL-2, IL-6 and IL-8 in DVT.

## INTRODUCTION

Venous thromboembolism (VTE) encompassing of deep vein thrombosis (DVT) and pulmonary embolism (PE) associates with considerable morbidity, functional disability and mortality [1, 2], and is the third most common cause of death in cardiovascular disease deaths after coronary heart disease and stroke [3]. The annual incidence of VTE of adult is more than 0.1% [4]. The incidence of DVT of the upper extremity, lower extremity and PE of children falls in a range of 0.07/10000 to 0.49/10000 [5]. A series of conditions are considered the risk factors of VTE. The spinal cord injury, the major surgery, the prolonged immobility, the bone fracture and the setting of the central catheters are the first class risk factors of DVT and PE [5–13]. The pancreas, ovarian, breast, lung and brain cancer, as well as the intracranial tumor surgery are the second class risk factors of DVT and PE [14–20]. Risk factors-associated DVT and PE have led to the poorer outcome and shorter survival of the patients [21, 22]. As a worldwide health problem the pathology, the pharmacology, the prophylaxis strategy and the therapy of DVT and PE have been extensively investigated. In the pathology investigation the activated platelets are considered to participate in each step of cancer- associated VTE [23], while inflammation is considered to implicate into VTE process either as a cause or as a consequence [24]. In the pharmacology investigation P-selectin [25, 26], GPIIb/IIIa [27], and some cytokines, such as IL-6 and IL-8 [28–30], have been clinically used as biomarker. In prophylaxis strategy investigation [31, 32], and therapeutic investigation [33], the efficacy and safety are particularly emphasized. However there still is no compound to associate the impact of down-regulating P-selectin, GPIIb/IIIa, IL-2, IL-6 and IL-8 on DVT therapy. In this context this paper analyzed the structural characteristics such as Arg-Gly-Asp-Val of anti-platelet aggregation agent aspirin-Arg-Gly-Asp-Val [34], dihydroxyl-tetrahydroisoquinoline-3-carboxylic acid of a P-selectin down-regulator dihydroxyl-tetrahydroisoquinoline-3-carbonyl-Lys-Pro-Ala-Lys [35], Thr-Ala-Arg-Gly-Asp(Ser)-Ser of a GPIIb/IIIa down-regulator N-(3S-1,2,3,4-tetrahydroisoquinoline-3-carbonyl)-Thr-Ala-Arg-Gly-Asp(Ser)-Ser [36], and tetrahydro-β-carboline-3-carboxylic acid of an anti-inflammatory agent tetrahydro-β-carboline-3-carboxyl-thymopentin [37] to design N-(3S-1,2,3,4-tetrahydroisoquinoline-3-carbonyl)-Thr-Ala-Arg-Gly-Asp(Val)-Val (IQCA-TAVV) as a novel inhibitor for essential evaluations and thereby to associate the contribution of the down- regulation of P-selectin, GPIIb/IIIa, IL-2, IL-6 and IL-8 to DVT inhibition.

## RESULTS

### Docking of IQCA-TAVV towards P-selectin and GPIIb/IIIa

**Figure 1:**
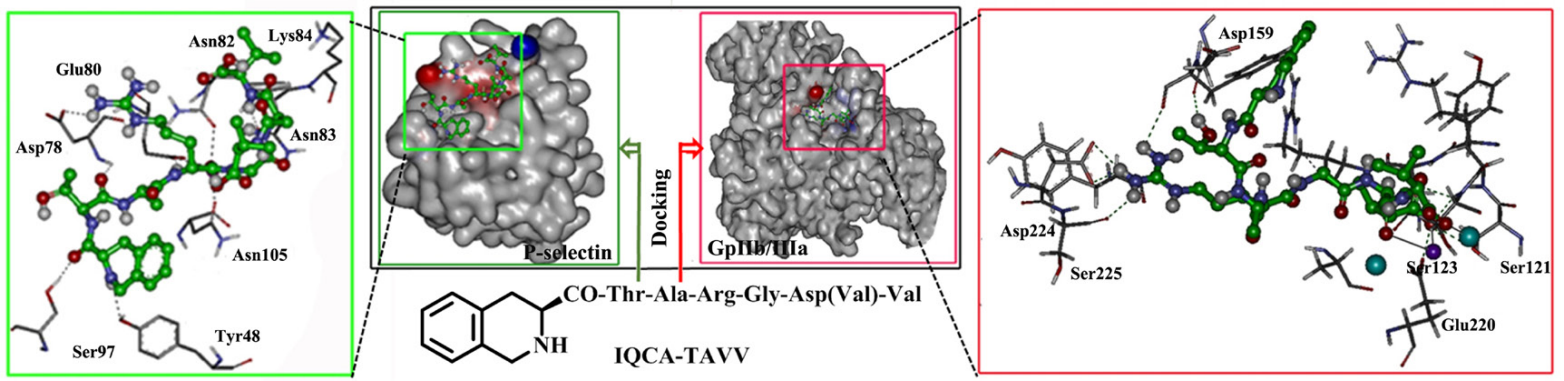
Docking features of IQCA-TAVV in the active site of P-selectin (green box) and GPIIb/IIIa (red box)

Docking study has been commonly used for the design of lead compounds. To theoretically predict the ability of IQCA-TAVV to target the active site of P-selectin (an average structure of PDB entry 1G1R via molecular dynamics simulations) and GPIIb/IIIa (PDB entry 2VDR) the docking investigation was performed. The green box of Figure 1 shows that IQCA-TAVV and 8 important residues Tyr48, Asp78, Glu80, Asn82, Asn83, Lys84, Ser97 and Asn105 of the active site form 11 hydrogen bonds. These hydrogen interactions lead the docking of IQCA-TAVV towards P-selectin having -7.03 kcal/mol of binding free energy. The red box of Figure 1 indicates that IQCA-TAVV and 7 important residues Ser121, Ser123, Asp159, Phe160, Glu220, Asp224 and Ser225 of the active site form 10 hydrogen bonds. Besides, IQCA-TAVV and the calcium ion and magnesium ion of active site form 2 coordinate bonds. These interactions lead the docking of IQCA-TAVV towards GPIIb/IIIa having -9.66 kcal/mol of binding free energy. Thus the docking features predict that IQCA-TAVV can properly target P-selectin and GPIIb/IIIa.

### Effect of IQCA-TAVV on the UV spectra of P-selectin and GPIIb/IIIa

**Figure 2:**
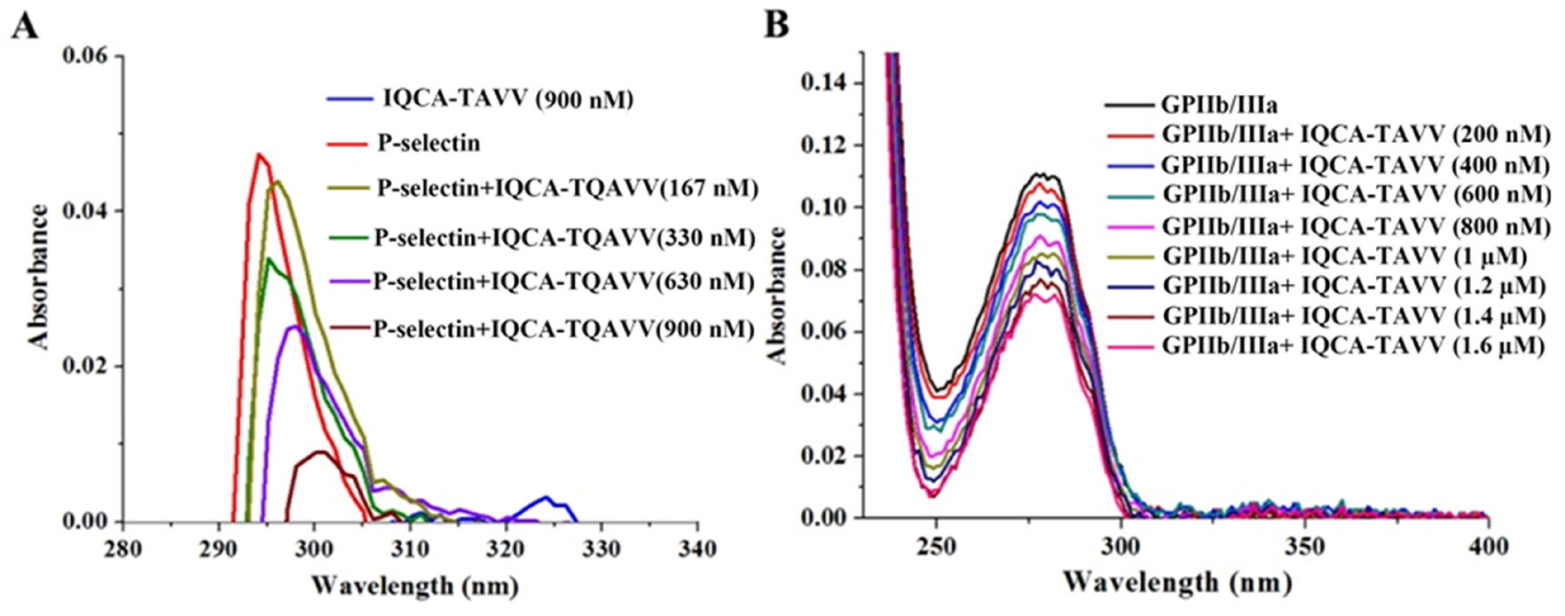
Effect of IQCA-TAVV on UV spectra of the solution of P-selectin and GPIIb/IIIa in sample diluents **(A)** UV spectra of IQCA-TAVV (final concentration: 900 nM), P-selectin (final concentration: 300 ng/mL), and P-selectin (final concentration: 300 ng/mL) plus IQCA-TAVV (final concentration: 167 nM-900 nM) in sample diluents; **(B)** UV spectra of GPIIb/IIIa (final concentration: 5.4 ng/mL) plus IQCA- TAVV (final concentration: 200 nM-1.6 mM) in sample diluents.

Figure 2A shows the UV spectra of the solution of P-selectin plus IQCA-TAVV in sample diluent, and indicates that the addition of IQCA-TAVV leads to the UV spectrum of P-selectin occurring hypochromic effect and red shift. The change of the UV spectrum in the maximal absorption wave length and the absorption intensity mirrors IQCA-TAVV binding P-selectin. Figure 2B shows the UV spectra of the solution of GPIIb/IIIa plus IQCA-TAVV in sample diluent, and indicates that the addition of IQCA-TAVV leads to the UV spectrum of GPIIb/IIIa occurring hypochromic effect and blue shift. The change of the UV spectrum in the maximal absorption wave length and the absorption intensity mirrors IQCA-TAVV binding GPIIb/IIIa. The dual actions of IQCA-TAVV on P-selectin and GPIIb/IIIa should benefit the anti-platelet aggregation activity, the anti-thrombotic activity and the thrombus target of IQCA-TAVV.

### Effect of IQCA-TAVV on the expression of P-selectin and GPIIb/IIIa

**Figure 3:**
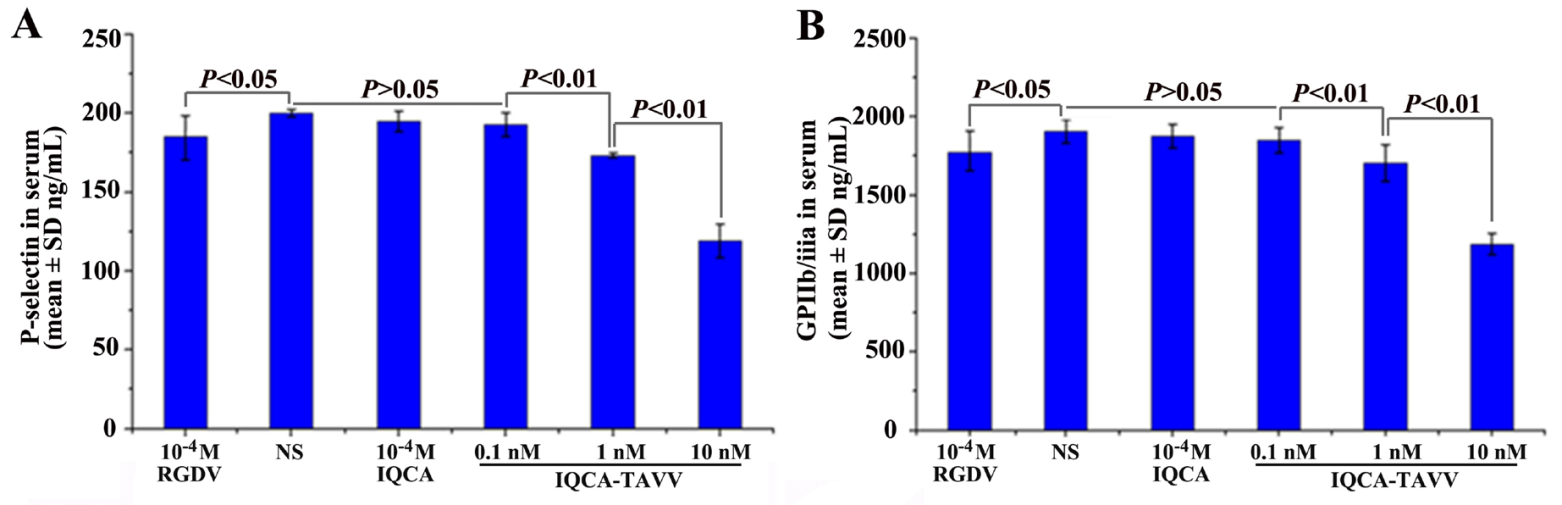
Effect of IQCA-TAVV on the expression of P-selectin and GPIIb/IIIa, n=6 **(A)** IQCA-TAVV concentration-dependently down-regulates P-selectin expression; **(B)** IQCA-TAVV concentration-dependently down-regulates GPIIb/IIIa expression.

The docking prediction was also confirmed with ELISA experiment. Figure 3A shows that IQCA-TAVV concentration dependently down-regulates arachidonic acid (AA) activated rat platelets to express P-selectin, and the minimal effective concentration is 1 nM. Figure 3B shows that IQCA-TAVV concentration dependently down-regulates AA activated rat platelets to express GPIIb/IIIa, and the minimal effective concentration is 1 nM. The concentrations of P-selectin and GPIIb/IIIa expressed by AA activated rat platelets with 1 nM of IQCA-TAVV are significantly lower than those of P-selectin and GPIIb/IIIa expressed by AA activated rat platelets with 10^-4^ M of IQCA. This means that the activity of IQCA-TAVV down-regulating AA activated rat platelets to express P-selectin and GPIIb/IIIa is 100000 folds higher than that of IQCA.

### Effect of IQCA-TAVV on platelet aggregation *in vitro*

**Figure 4:**
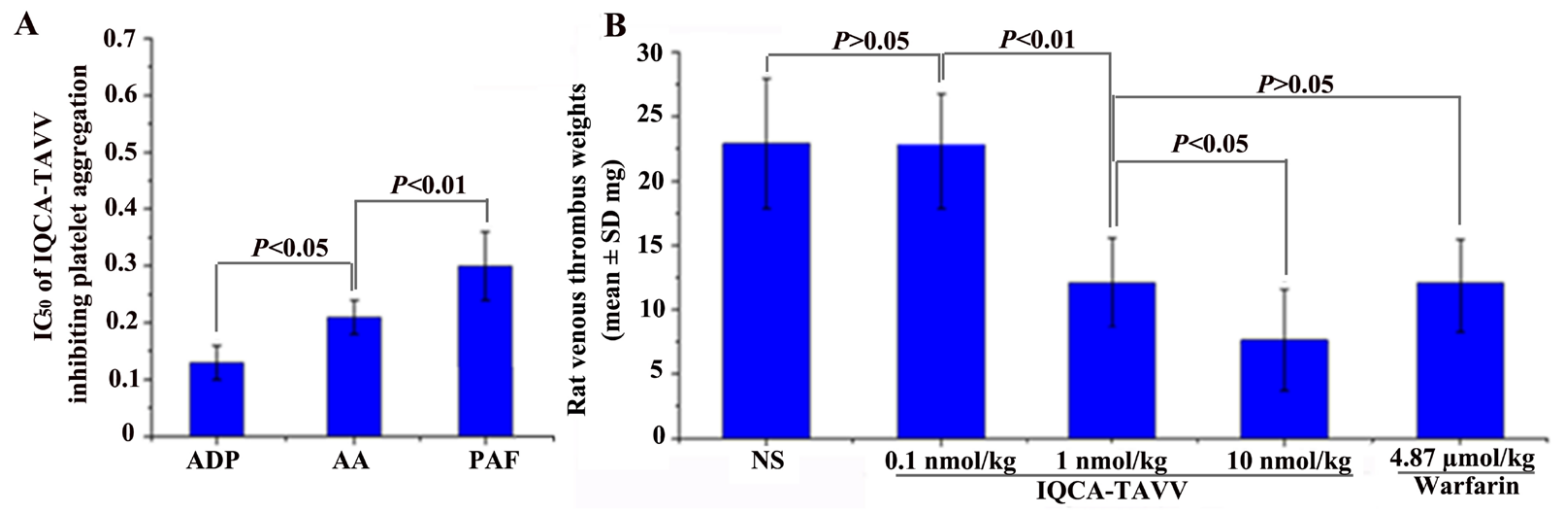
Effects of IQCA-TAVV on platelet aggregation and venous thrombosis **(A)** *In vitro* IQCA-TAVV effectively inhibits rat platelet aggregation, n=6; **(B)** *In vivo* IQCA-TAVV dose-dependently inhibits the rats to form venous thrombus, n=12.

As mentioned above the platelet activation is of critically importance for venous thrombosis. To estimate the value of IQCA-TAVV as the thrombotic inhibitor the *in vitro* anti-platelet aggregation assays were performed. Figure 4A indicates that the IC_50_ values of IQCA-TAVV inhibiting rat platelet aggregation induced by AA (final concentration 350 μM), adenosine diphosphate (ADP, final concentration 10 μM) and platelet activating factor (PAF, final concentration 0.1 μM) are 0.21 nM, 0.13 nM and 0.30 nM, respectively. The IC_50_ values fall within a range of 0.13 nM to 0.30 nM means that IQCA-TAVV is a powerful anti-platelet aggregation agent. Of the three aggregators ADP is the most sensitive aggregator to IQCA-TAVV.

### Effect of IQCA-TAVV on venous thrombosis *in vivo*

The *in vivo* activity of inhibiting venous thrombosis is an important issue for a DVT inhibitor. To see if IQCA-TAVV been a valid DTV inhibitor the rat venous thrombosis model was used to test the venous thrombus weight. Figure 4B indicates that oral IQCA-TAVV dose dependently inhibits the rats to form venous thrombus. The venous thrombus weight of the rats treated with 1 nmol/kg of IQCA-TAVV is significantly lower than that of the rats treated with NS and is equal to that of the rats treated with 4.87 μmol/kg of warfarin. The comparison suggests that the minimal effective dose of IQCA-TAVV to inhibit venous thrombosis is 1 nmol/kg. The comparison further suggests that the activity of IQCA-TAVV is 4870 folds higher than that of warfarin. Thus IQCA- TAVV is a powerful DVT inhibitor.

### Effect of IQCA-TAVV on inflammation *in vivo*

**Figure 5:**
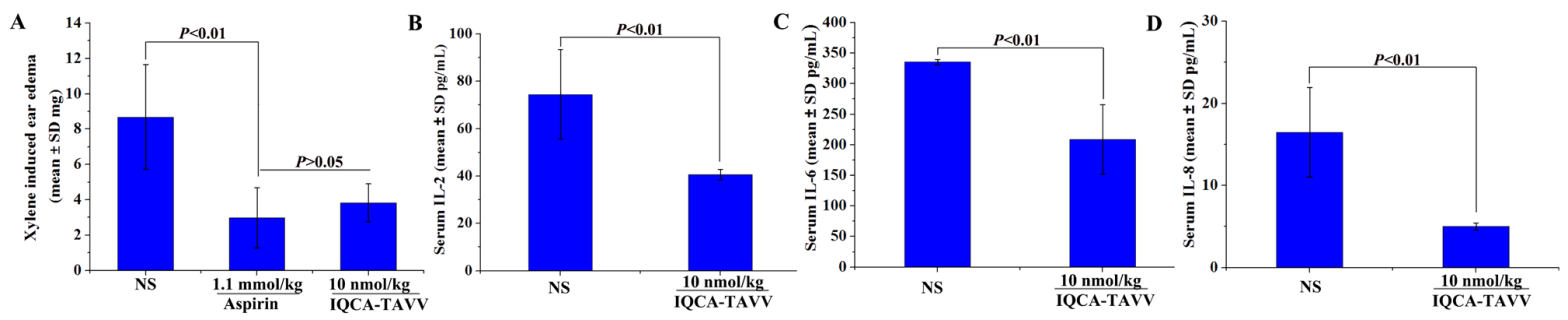
Effect of IQCA-TAVV on inflammation, IL-2, IL-6 and IL-8 **(A)** *In vivo* anti-inflammatory activity of IQCA-TAVV, n=12; **(B)** Serum IL-2 of the inflammatory mice treated with IQCA-TAVV, n = 12; **(C)** Serum IL-6 of the inflammatory mice treated with IQCA-TAVV, n = 12; **(D)** Serum IL-8 of the inflammatory mice treated with IQCA-TAVV, n = 12.

As mentioned above, inflammation is implicated in VTE either as a cause or as a consequence. To explore the effect of IQCA-TAVV on inflammation the xylene-induced ear edema model of mice was practiced. Figure 5A shows that the ear edema of the mice orally treated with 10 nmol/kg of IQCA-TAVV is significantly lower than that of the mice treated with NS, and is equal to that of the mice treated with 1.1 mmol/kg of aspirin. The comparison suggests that the anti-inflammation activity of IQCA-TAVV is 110000 folds higher than that of aspirin. Therefore IQCA-TAVV is a powerful inflammatory inhibitor.

### Effect of IQCA-TAVV on IL-2, IL-6 and IL-8 in the serum of inflammatory mice

Some cytokines, such as IL-6 and IL-8, are not only highly amenable to serve as the diagnostic indicator of inflammation but also highly amenable to serve as the predictor the progress of DVT. To estimate the effect of IQCA-TAVV on cytokines the concentration of IL-2, IL-6 and IL-8 in the serum of the mice receiving xylene-induced ear edema assay were measured. Figures 5B, 5C and 5D indicate that at 10 nmol/kg dose of IQCA-TAVV effectively decreases the concentration of IL-2, IL-6 and IL-8 in the serum of the mice. The data imply that IQCA-TAVV is able to block the progress of DVT.

### Acute toxicity of IQCA-TAVV treated mice

**Figure 6:**
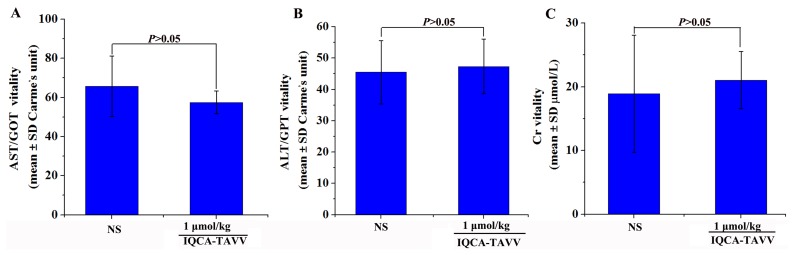
Effects of IQCA-TAVV on mouse Cr, ALT and AST **(A)** Serum Cr of ICR mice treated with NS and 1 μmol/kg of IQCA-TAVV (n=5); **(B)** Serum ALT of ICR mice treated with NS and 1 μmol/kg of IQCA-TAVV (n=5); **(C)** Serum AST of ICR mice treated with NS and 1 μmol/kg of IQCA-TAVV (n=5).

To emphasize the therapeutic safety the healthy mice were treated with 1 μmol/kg (100-1000 folds of the minimal effective dose) of IQCA-TAVV to observe neurotoxicity, liver toxicity and kidney toxicity. It was found that even receive such a high dose the mice neither exhibited neurotoxicity behavior, such as tremor, twitch, jumping, tetanus, and supination, nor occurred death. This suggests that the LD_50_ value of IQCA-TAVV is more than 1 μmol/kg. Figure 6A, 6B and 6C indicate that the concentration of alanine transaminase (ALT), aspartate transaminase (AST) and creatinine (Cr) in the serum of the mice treated with 1 μmol/kg of IQCA-TAVV are equal to those of the mice treated with NS. All the observations together suggest that at such a high dose IQCA- TAVV induces low neurotoxicity and does not injure the kidney and the liver.

## DISCUSSION

DVT is the third most common cause of death in cardiovascular disease deaths after coronary heart disease and stroke. The activation of platelets participates in each step of DVT progress, while inflammation is implicated into DVT process either as a cause or as a consequence. The impact of platelet activation and inflammatory response on the onset of DVT implies that the inhibition of platelet aggregation, the down-regulation of the expression of P-selectin and GPIIb/IIIa, the decrease of serum levels of IL-2, IL-6 and IL-8 can benefit the prophylaxis of DVT. Consequently these pharmacology efficacies and the association thereof can be recognized with an inhibitor. For this purpose, the structural elements in an anti-platelet aggregation agent, a P-selectin down-regulator, a GPIIb/IIIa down-regulator and an anti-inflammatory agent were combined into IQCA-TAVV.

The docking feature shows that the energy minimized conformation of IQCA-TAVV can properly interact with 8 important residues of the active site of P-selectin to form 11 hydrogen bonds. The docking feature also shows that the energy minimized conformation of IQCA-TAVV can properly interact with 7 important residues and two ions of the active site of GPIIb/IIIa to form 10 hydrogen bonds and 2 coordinate bonds. Thus the docking predicted that IQCA-TAVV should be a desirable ligand of P-selectin and GPIIb/IIIa, the two important membrane receptors of platelets. From the perspective of the experiment these predictions could be confirmed by the expression of P-selectin and GPIIb/IIIa of the activated platelets.

To support the docking feature the amount of P-selectin and GPIIb/IIIa expressed by AA activated platelets treated with NS and IQCA-TAVV was identified. ELISA experiments indicated that IQCA-TAVV concentration dependently down-regulated the expression of P-selectin and GPIIb/IIIa. The minimal effective concentration as low as 1 nM shows that IQCA-TAVV is capable of targeting the externalized active site and consequently is capable of inhibiting the expression of P-selectin and GPIIb/IIIa. This capacity mirrors that IQCA-TAVV can block P-selectin and GPIIb/IIIa been sheared from the membrane of the activated platelets. It is in the activated platelets, but not in the resting platelets, P-selectin and GPIIb/IIIa exist in the externalized form, but not in the internalized form.

The inhibition of AA, ADP and PAF was measured with anti-platelet aggregation experiments. *in vitro* IQCA-TAVV effectively inhibited AA, ADP and PAF induced platelet aggregation, and the IC_50_ values ranged from 0.13 nM to 0.30 nM. These IC_50_ values emphasize that IQCA-TAVV is a powerful inhibitor of AA, ADP and PAF. Consequently IQCA-TAVV is a powerful inhibitor of the expression of P-selectin and GPIIb/IIIa.

The *in vitro* anti-platelet aggregation was correlated with the *in vivo* anti-venous thrombosis. On inferior vena cava ligation model the venous thrombus weight of the rats treated with 1 nmol/kg of IQCA-TAVV is equal to that of the rats treated with 4.87 μmol/kg of warfarin. This suggests that the activity of IQCA-TAVV is 4870 folds higher than that of warfarin. IQCA-TAVV could be a higher effective anti-venous thrombosis agent.

The anti-thromboinflammatory strategy is considered a future strategy of DVT prophylaxis [38]. To practice this strategy here the anti-inflammatory activity of IQCA-TAVV was evaluated. On the xylene-induced ear edema model the ear edema of the mice treated with 10 nmol/kg IQCA-TAVV was equal to that of the mice treated with 1.1 mmol/kg aspirin. The anti-inflammatory activity of IQCA-TAVV is 110000 folds higher than that of aspirin. IQCA-TAVV is a potentially effective anti-inflammatory agent, and can suitably serve the higher effective anti-thromboinflammatory strategy for further DVT prophylaxis.

Cytokines IL-6 and IL-8 are the diagnostic indicators of inflammation and DVT. Both IL-2 and P-selectin mediate CD8^+^ T cells [39, 40], and both IL-2 and GPIIb/IIIa are involved in the immune thrombocytopenia related CD8^+^ T cells [41]. These correlations led to the measurement of IL-2, IL-6 and IL-8 in the serum of the mice receiving xylene-induced ear edema assay. At 10 nmol/kg of dose IQCA-TAVV effectively lowered the concentration of IL-2, IL-6 and IL-8 in the serum of the mice. IQCA-TAVV is a potential down-regulator of IL-2, IL-6 and IL-8.

To emphasize the therapeutic safety of IQCA-TAVV its dose was raised to 1 μmol/kg, a dose of 100-1000 folds of its minimal effective dose (1 - 10 nmol/kg). It was found that even giving such a high dose the healthy mice neither exhibited neurotoxicity nor occurred death. This means that the therapy of IQCA-TAVV has a very large safe window.

Growing findings evidence that the inhibition of platelet activation results in the block of the externalization and the expression of P-selectin and GPIIb/IIIa [42–44], results in the inhibition of the inflammatory response [43, 45, 46], and results in the down-regulation of IL-2, IL-6 and IL-8 [39–41]. In consistent with these findings, the evaluations herein explore that by inhibiting platelet activation IQCA-TAVV simultaneously down-regulates P-selectin, GPIIb/IIIa, IL-2, IL-6 and IL-8. Obviously, the inhibition of platelet activation is a key event of IQCA-TAVV depressing P-selectin and GPIIb/IIIa expression, attenuating inflammatory response and thereby decreasing the content of IL-2, IL-6 and IL-8 in the serum of inflammatory mice. IQCA-TAVV also shows that to block the expression of the activated platelets simultaneously targeting the externalized P-selectin and GPIIb/IIIa is of critical importance. Thus IQCA-TAVV depicts a relationship of three levels (from inhibiting platelet activation to targeting externalized membrane receptor and to decreasing serum inflammatory factor) for the down-regulation of P-selectin, GPIIb/IIIa, IL-2, IL-6 and IL-8 in the therapy of DVT.

## MATERIALS AND METHODS

### General

All chemicals were commercially available without further purification. Chromatography was carried out on silica gel H (Qingdao Chemical Factory, China). IQCA-TAVV was prepared ourselves. The analytical data of IQCA-TAVV were HPLC (waters, C_18_ column, 4.6×150 mm) and was more than 99%; ESI-MS (m/e) 874 [M - H]^-^, Mp 164 - 165 °C, [α]_D_^25^ = -10.1 (c = 1.4, CH_3_OH); IR (cm^-1^) 3446, 2963, 2386, 1653, 1542, 1456, 1390, 1254, 1172, 1029, 762, 641, 515, 437; ^1^H NMR (800 MHz, DMSO*-d6*) δ/ppm = 8.36 (m, 1 H), 8.20 (m, 1 H), 8.08 (m, 1H), 8.03 (m,1H), 7.98 (m, 1 H),7.94 (m, 1 H), 7.82 (m, 1 H), 7.20 (m, 4 H), 5.02 (br, 1 H), 4.62 (m,1 H), 4.31 (m, 2 H), 4.27 (m, 2 H), 4.13 (m, 2 H), 3.72 (m, 1 H), 3.08 (m, 2 H), 2.88 (dd, *J* = 3.2 Hz, *J* = 4.8 Hz, 1 H), 2.58 (dd, *J* = 3.2 Hz, *J* = 4.8 Hz, 1 H), 1.71 (m, 1 H), 1.54 (m, 1 H), 1.50 (m, 2 H), 1.26 (t, *J* = 7.2 Hz, 3 H), 1.09 (dd, *J* = 5.6 Hz, *J* = 6.4 Hz 3 H). 0.85 (m, 12 H); ^13^C NMR (200 MHz, DMSO*-d6*) δ/ppm = 172.53, 172.46, 171.51, 170.89, 170.69, 168.80, 168.69, 167.98, 167.85, 156.15, 134.87, 131.15, 128.08, 126.37, 125.84, 125.62, 66.11, 64.30, 61.95, 57.42, 56.99, 56.68, 54.21, 51.63, 47.73, 41.23, 36.55, 29.49, 29.37, 28.63, 19.02, 18.48, 17.49.

Male Sprague Dawley rats and ICR mice were purchased from the Animal Center of Capital Medical University. Animal experiments were reviewed and approved by the ethics committee of Capital Medical University. The committee assures that the welfare of the animals was maintained in accordance to the requirements of the Animal Welfare Act and in accordance to the NIH Guide for Care and Use of Laboratory Animals. Test and ANOVA were used for statistical analyses of all data. *P*-values < 0.05 were considered statistically significant.

### Generation of 3D structure of IQCA-TAVV for docking

The 2D structure of IQCA-TAVV was sketched in ChemDraw Ultra 10.0, converted to 3D conformation in Chem3D 10.0, and then energy was minimized until the minimum RMS to reach 0.001 in Chem3D Ultra 10.0. The energy optimized conformations in the whole conformational space of IQCA-TAVV were sampled with systematic search and BEST method of Discovery Studio 3.5, which were practiced with a SMART minimizer using CHARMM force field. The energy threshold at 300 K was 20 kcal/mol. The maximum minimization steps were 200 and the minimization root mean squared (RMS) gradient was 0.1 Å. The maximum generated conformations were 255 with a RMS deviation (RMSD) cutoff of 0.2 Å. The lowest energy conformation of IQCA-TAVV was used for the docking to the active site of P-selectin and GPIIb/IIIa.

### Docking of IQCA-TAVV into the active site of P-selectin

Software AutoDock 4 was used to perform the docking of IQCA-TAVV toward the active site of P-selectin. The average structure of P-selectin was obtained from the molecular dynamics simulations of PDB entry 1G1R. P-selectin was treated as rigid and prepared with AutoDockTools 1.5 by merging nonpolar hydrogens, assigning gasteiger charges and assigning autodock elements. IQCA-TAVV was treated as rigid and prepared with AutoDockTools 1.5 by merging nonpolar hydrogens, assigning gasteiger charges, finding root and aromatic carbons, detecting rotatable bonds and setting torsions. The grid box dimensions were 50 Å × 70 Å × 60 Å with a grid spacing of 0.375 Å. The grid box center was the Ca^2+^ in the crystal structure PDB 1G1R. Lamarckian genetic algorithm (LGA) was used to find the appropriate binding positions, orientations, and the conformations of IQCA-TAVV in the active site of P-selectin. The global optimization was started with the parameters of 300 randomly positioned individuals. The maximum number of energy evaluations was increased to 2.5 × 10^7^, and the maximum number of generations in LGA was increased to 2.7 × 10^5^. Solis and Wets local search was performed with a maximum number of 3000. During docking experiments 200 runs were carried out for IQCA- TAVV. The resulted 200 conformations of IQCA-TAVV were scored by the lowest binding energy and clustered with an rms tolerance of 2.0 Å.

### Docking of IQCA-TAVV into the active site of GPIIb/IIIa

Software AutoDock 4 was used to perform the docking of IQCA-TAVV toward the active site of GPIIb/IIIa (PDB entry 2VDR). GPIIb/IIIa was treated as rigid and prepared with AutoDockTools 1.5 by merging nonpolar hydrogens, assigning gasteiger charges and assigning autodock elements. IQCA-TAVV was treated as rigid and prepared with AutoDockTools 1.5 by merging nonpolar hydrogens, assigning gasteiger charges, finding root and aromatic carbons, detecting rotatable bonds and setting torsions. The grid box dimensions were 80 Å × 80 Å × 80 Å with a grid spacing of 0.375 Å. The grid box center was the center of the ligand in the crystal structure PDB 2VDR. LGA was used to find the appropriate binding positions, orientations and the conformations of IQCA-TAVV in the active site of GPIIb/IIIa. The global optimization was started with parameters of 300 randomly positioned individuals. The maximum number of energy evaluations was increased to 2.5 × 10^7^, and the maximum number of generations in LGA was increased to 2.7 × 10^5^. Solis and Wets local search was performed with a maximum number of 3000. During docking experiments 200 runs were carried out for IQCA-TAVV. The resulted 200 conformations of IQCA-TAVV were scored by the lowest binding energy and clustered with an rms tolerance of 2.0 Å.

### Addressing action of IQCA-TAVV on P-selectin *in vitro*

P-Selectin and sample diluents were commercially obtained from rat ELISA kit (Cusabio Biotech Co. Ltd, Newark, USA). A stock solution of IQCA-TAVV in sample diluents (4.38 mM) was prepared. A solution of P-selectin in sample diluents was prepared (300 ng/mL), from which 300 μL was added into an eppendorf tube. The control tube only contains 300 μL of diluents. The sample tube contains 300 μL solution of P-selectin in sample diluents (300 ng/mL) and 10 μL solution of IQCA-TAVV in sample diluents (final concentration: 167 nM, 330 nM, 630 nM and 900 nM). All tubes were incubated at room temperature for 12 h and then received UV test on a Shimadzu 2550 spectrophotometer.

### Addressing action of IQCA-TAVV on GPIIb/IIIa *in vitro*

GPIIb/IIIa and sample diluents were commercially obtained from rat ELISA kit (Cusabio Biotech Co. Ltd, Newark, USA). A stock solution of IQCA-TAVV in sample diluents (4.38 mM) was prepared. A solution of GPIIb/IIIa in sample diluents was prepared (5.4 ng/mL), from which 300 μL was added into six eppendorf tubes. The control tube only contains 300 μL of diluents. The sample tube contains 300 μL solution of GPIIb/IIIa in sample diluents (5.4 ng/mL) and 10 μL solution of IQCA-TAVV in sample diluents (final concentration: 200 nM, 400 nM, 600 nM, 800 nM, 1.0 μM, 1.2 μM, 1.4 μM and 1.6 μM,). All tubes were incubated at room temperature for 12 h and then received UV test on a Shimadzu 2550 spectrophotometer.

### ELISA based P-selectin assay of IQCA-TAVV

Rat blood was collected into a tube containing 3.8% sodium citrate at a ratio of 9/1 and immediately centrifuged at 1000 rpm for 15 min. The top layer was collected as platelet-rich plasma (PRP), which received 10-fold-dilution with diluents solution (from the kit) to get PRP sample. P-Selectin levels of activated platelets with and without IQCA-TAVV were measured by use of rat P-selectin ELISA kit (Cusabio, Biotech, USA). To 980 μL of PRP 10 μL of NS or IQCA-TAVV solution (final concentration, 10 nM) in NS was added, and then 10 μL of AA solution 0.15 mg/mL) was added, at 37 °C incubated for 3 min to prepare the blank solution or IQCA-TAVV solution. To the control well and the test well of a 96-well plate coated with the enzyme, 100 μL blank solution or IQCA-TAVV solution were added, respectively. The wells were at 37 °C incubated for 120 min. After removing the solvent, 100 μL of biotin labeling antibody (from the kit) was added to each well and then at 37 °C incubated for 60 min. The solution of each well was discarded, 200 μL of washing solution (from the kit) was added to wash the well for three times. After adding 100 μL/well of horseradish peroxidase labeling avidin (from the kit), the plate was at 37 °C incubated for 60 min, and then washed five times. For coloration, each well received 90 μL of the substrate solution (from the kit) and then in dark at 37 °C incubated for 20 min. To stop the reaction 50 μL/well of the stop solution (from the kit) was added. The OD value of the well was measured at 450 nm within 15 min. Consequently P-selectin level was calculated by using the standard curve (from the kit standard samples).

### ELISA based GPIIb/IIIa assay of IQCA-TAVV

Rat blood was collected into a tube containing 3.8% sodium citrate at a ratio of 9/1 and immediately centrifuged at 1000 rpm for 15 min. The top layer was collected as platelet-rich plasma (PRP), which received 10-fold-dilution with diluents solution (from the kit) to get PRP sample. GPIIb/IIIa levels of activated platelets with and without IQCA-TAVV were measured by use of rat GPIIb/IIIa ELISA kit (Cusabio Biotech Co. Ltd, Newark, USA). To 980 μL of PRP 10 μL of NS or IQCA-TAVV solution (final concentration, 10 nM) in NS was added, and then 20 μL of AA solution 0.15 mg/mL) was added, at 37 °C incubated for 5 min to prepare the blank solution or IQCA-TAVV solution. To the control well and the test well of a 96-well plate coated with the enzyme, 100 μL blank solution or IQCA-TAVV solution were added, respectively. The wells were at 37 °C incubated for 30 min. After removing the solvent, 50 μL of biotin labeling antibody (from the kit) was added to each well and then at 37 °C incubated for 30 min. The wells were washed with washing solution (from the kit) for five times, into which 50 μL chromogen solution A and B were successively added, at 37 °C in dark colored for 15 min, and then 50 μL of stop solution was added to stop the coloration. The OD value of the well was measured at 450 nm within 15 min. Consequently GPIIb/IIIa concentration was calculated by using the standard curve (from the kit standard samples).

### *In vitro* platelet aggregation assay of IQCA-TAVV

Platelet aggregation was evaluated with two-channel Chronolog aggregometer by following manufacturer’s instructions. The citrated pig blood was immediately centrifuged at 1000 rpm for 15 min to get PRP and further centrifuged at 3000 rpm for another 10 min to get platelet poor plasma (PPP). With stirring in an optical glass cuvette 500 μL PRP was diluted with PPP and the concentration of the platelets became to ∼2×10^8^ platelets/mL, into which 5 μL NS or 5 μL solution of IQCA-TAVV in NS (final concentrations ranging from 0.01 nM to 10 nM) was added. After adjustment of the baseline, 5 μL solution of platelet activating factor (PAF) in NS (final concentration 0.1 μM) or 5μL solution of adenosine diphosphate (ADP) in NS (final concentration 10 μM) or 5 μL solution of arachidonic acid (AA) in NS (final concentration 350 μM) was added. At 37 °C the change of the light transmission was measured for 5min, and the activity of IQCA-TAVV against the aggregation induced by PAF or ADP or AA were recorded. The inhibition rate was calculated by the following inhibition%= [1 - (A_m_% of IQCA-TAVV)/(A_m_% of NS)]×100%, wherein Am% was the maximal rate of platelet aggregation and represented by the peak height of aggregation curve. IC_50_ values were obtained from dose-response curves and calculated with a logarithmic curve fitting program.

### *In vivo* rat venous thrombosis assay of IQCA-TAVV

An inferior vena cava (IVC) ligation model was used to evaluate the venous thrombosis. In brief, male Sprague Dawley rats (200 ± 20 g) were randomly divided into 5 groups of 12 animals each for orally receiving IQCA-TAVV (0.1, 1 and 10 nmol/kg), or warfarin (4.87 μmol/kg), or NS (2 mL/kg). Thirty min after the administration the rat was anaesthetized with sodium pentobarbital (80.0 mg/kg, ip), and then a midline laparotomy was performed. The small bowel was exteriorized from the body cavity and moved slightly to the left of the animal surrounding moistened gauze, the infrarenal IVC was separated, the junction of the IVC and left renal vein on the IVC was ligated with braided black silk suture of 4/0. In addition, warm saline was sprayed, the tissues backed to the abdominal cavity, muscle layer and skin were sutured with braided black silk suture of 4/0. Four hours later, the abdominal cavity was reopened, the ligatures were applied near the bifurcation of the IVC and around all side branches of the ligated IVC segment. Consequently, 2 cm of venous segment bellow the ligation site was excised, the thrombus was removed, the blood was blotted with filter paper, and the thrombus was weighed to represent the activity.

### *In vivo* xylene-induced ear edema assay of IQCA-TAVV

Male ICR mice (25 ± 2 g) were randomly divided into 3 groups of 12 animals each for orally receiving IQCA-TAVV (10 nmol/kg), or aspirin (1.1 mmol/kg), or NS (10 mL/kg). Onto the anterior and posterior surfaces of the right ear of each mouse 30 μL of xylene was applied, while the left ear without xylene was used as blank control. Two hours after xylene application, the mice were anesthetized with ether and sacrificed to collect the citrated whole blood and to remove of both ears. The citrated blood was immediately centrifuged at 1000 rpm for 15 min to give PRP. The left and right ears of each mouse were punched with a cork borer of 7 mm in diameter to get circular pieces for weighing the difference between them to address the degree of ear swelling and represent the activity.

### Measuring serum IL-2, IL-6 and IL-8 of IQCA-TAVV treated inflammatory mice

The blood obtained from the mice receiving *in vivo* xylene-induced ear edema assay was centrifuged at 3000 rpm for 15 min to get the serum. Serum IL-2, IL-6 and IL-8 were measured by using enzyme- linked immunosorbent kit (ELISA, BioSource, Europe SA, 8 B-1400, Nivelles, Belgium). In brief, to each of three blank wells 100 μL diluents was added. To each of six standard wells 50 μL standard solution and 50 μL streptavidin-HRP were added. To each of three testing wells 40 μL serum from inflammatory mice treated by NS or IQCA-TAVV (10 nmol/kg), 10 μL anti-IL-2-antibody, 10 μL anti-IL-6-antibody or 10 μL anti-IL-8-antibody and 50 μL streptavidin-HRP were successively added. The plate was covered with closure membrane and at 37 °C incubated for 60 min. On uncovering the membrane the liquid in the well was discarded and dried by swing. The residue in the well was mixed with sufficient washing buffer, stired for 30 s and draied. The washing buffer was prepared by mixing the wash solution and distilled water in a volume ratio of 1/30. The washing procedure was repeated for 5 times, the well was dried by pat. Into the well having residue 50 μL chromogen solution A and B were added, gently mixed and in dark at 37 °C incubated for 10 min. Into the well 50 μL stop solution was added, and the color in the well changed from blue to yellow. At 450 nm the plate was read with microtiter plate reader within 15 min to record the O.D. value. With the standard curve the serum IL-2 or IL-6 or IL-8 was calculated.

## CONCLUSIONS

IQCA-TAVV is a novel venous thrombosis inhibitor. The pharmacology profile of IQCA-TAVV includes the docking towards the active sites of P-eslectin and GPIIb/IIIa, the binding to P-slectin and GPIIb/IIIa, the depression of activated platelets to express P-slectin and GPIIb/IIIa, the block of AA, ADP and PAF to induce platelet aggregation, the decrease of venous thrombus weight of the rats, the remission of ear edema of inflammatory mice, and the decrease of the levels of IL-2, IL-6 and IL-8 in the serum of inflammatory mice. Thereby IQCA-TAVV depicts a relationship of three levels (from inhibiting platelet activation to targeting externalized membrane receptor and to decreasing serum inflammatory factor) for the down-regulation of P-selectin, GPIIb/IIIa, IL-2, IL-6 and IL-8 in the therapy of DVT. With the finding that even the dose been 100 -1000 folds of its minimal effective dose IQCA-TAVV still induces no any toxicity reaction together accumulate evidences are provided for IQCA-TAVV to serve the higher effective anti-thromboinflammatory strategy of treating DVT.
